# Distinguish the Severity of Illness Associated with Novel Coronavirus (COVID-19) Infection via Sustained Vowel Speech Features

**DOI:** 10.3390/ijerph20043415

**Published:** 2023-02-15

**Authors:** Yasuhiro Omiya, Daisuke Mizuguchi, Shinichi Tokuno

**Affiliations:** 1 PST Inc., Yokohama 231-0023, Japan; 2 Department of Bioengineering, Graduate School of Engineering, The University of Tokyo, Tokyo 113-8656, Japan; 3 Graduate School of Health Innovation, Kanagawa University of Human Services, Yokosuka 210-0821, Japan

**Keywords:** voice biomarker, COVID-19, sustained vowel

## Abstract

The authors are currently conducting research on methods to estimate psychiatric and neurological disorders from a voice by focusing on the features of speech. It is empirically known that numerous psychosomatic symptoms appear in voice biomarkers; in this study, we examined the effectiveness of distinguishing changes in the symptoms associated with novel coronavirus infection using speech features. Multiple speech features were extracted from the voice recordings, and, as a countermeasure against overfitting, we selected features using statistical analysis and feature selection methods utilizing pseudo data and built and verified machine learning algorithm models using LightGBM. Applying 5-fold cross-validation, and using three types of sustained vowel sounds of /Ah/, /Eh/, and /Uh/, we achieved a high performance (accuracy and AUC) of over 88% in distinguishing “asymptomatic or mild illness (symptoms)” and “moderate illness 1 (symptoms)”. Accordingly, the results suggest that the proposed index using voice (speech features) can likely be used in distinguishing the symptoms associated with novel coronavirus infection.

## 1. Introduction

The novel coronavirus (COVID-19) is spreading worldwide, and, in some countries and regions, individuals infected with COVID-19 are forced to recuperate at home, isolated from the outside world, with restrictions on contact with others. Percutaneous oxygen saturation (SpO_2_) measured by a pulse oximeter is used as one of the objective criteria for determining the worsening of the symptoms associated with COVID-19 infection in patients recuperating at home.

The severity of COVID-19 patients in Japan is classified into four stages, as shown in Table 1, and moderate or severe symptoms require treatment at a medical institution [1]. Accordingly, distinguishing between moderate and mild symptoms is extremely important because patients, upon becoming moderately ill while recuperating at home, may need to be transported to a medical institution. As observed in Table 1, the SpO_2_ level measured using a pulse oximeter is a key objective index for distinguishing between the stages.

However, it is difficult to provide pulse oximeters to all patients due to their huge number. This calls for a simple symptom monitoring technology that can replace a pulse oximeter. As an example of the effectiveness of vocal biomarkers in estimating symptoms and diseases, we have reported on the differentiation of stress and mental illness using voice [2,3]. Herein, [3] reported a significant negative correlation between voice indicators and psychological test (Hamilton Rating Scale for Depression) scores (r = −0.33, *p* < 0.05), and the voice indicator was able to discriminate between healthy and depressed speech data with high accuracy (*p* = 0.0085, area under the receiver operating characteristic curve = 0.76). Thus, vocal biomarkers might also be applied to distinguish changes in the symptoms of COVID-19.

Accordingly, we focused on the changes in voice associated with respiratory distress. An analysis using voice has the advantage that it can be performed easily and remotely and is effective for monitoring and screening.

As for related research, studies have been conducted on detecting COVID-19 infection using cough, sustained vowel sound, questions, or a combination of them [4,5,6,7,8,9,10]. They are intended to determine whether or not the participants are infected with COVID-19, but not to judge if they need medical intervention due to the increase in severity of their symptoms, which is the subject of this study.

Further, a smartphone device application was used to collect participants’ multiple cough and vowel /Ah/ recordings to estimate positive or negative COVID-19 status, using many acoustic features, such as openSMILE, PRAAT, LIBROSA, and feature set based on a D-CNN model [4]. Some of the audio features overlap with our study, but the results use the majority voting per-day method for both /Ah/ vowels and coughs, achieving 0.69 and 0.74 AUC scores, respectively. The target is different from our study and a further improvement in accuracy is required.

In another recent study [5], they used crowdsourced respiratory audio data, including breathing, cough, and voice, collected from each participant over a period of time, together with self-reported COVID-19 test results, using audio sequence longitudinally with Deep Neural Network (Gated Recurrent Units) techniques, achieving AUC scores of 0.74–0.84, sensitivity of 0.67–0.82, and specificity of 0.67–0.75. They claim that time-series data of audio increase the accuracy of COVID-19 detection and monitoring for disease progression, especially the recovery trajectory of individuals to be more effective in monitoring recovery than single audio, but the assumption must be made that sequential audio is being recorded, and a further improvement in accuracy is required.

In this study, we examined the effectiveness of distinguishing the changes in the symptoms associated with COVID-19 infection by voice using speech features. Specifically, the purpose was to propose a model using voice to accurately distinguish mild illness and moderate illness I in patients with COVID-19 infection. We collected voice recording from COVID-19-positive patients during the periods when Japan experienced the spread of the Delta and Omicron variants (the so-called “5th wave” and “6th wave”, respectively) [11,12]. For the mild illness and moderate illness I groups, we created a data set that matched age and sex before the 5th wave and after the 6th wave and examined it using cross-validation. We extracted openSMILE features [13] from the voice data of the participants, and, as a countermeasure against overfitting, we selected features using correlation coefficients and null importance [14], which tests actual feature importance against the distribution of those when fitted to the shuffled target. Subsequently, we built and validated the machine learning (ML) algorithm using LightGBM [15].

## 2. Method

### 2.1. Ethical Considerations

This study was approved by the Research Ethics Review Committee, Kanagawa University of Health and Welfare (Approval No. SHI 3-001).

### 2.2. Data Collection

The subjects in this study were COVID-19-positive patients aged 20 years or older who were judged to require recuperation at lodging facilities or home. Recruitment pamphlets for participation in the research were distributed to COVID-19-infected patients in Kanagawa prefecture between June 2021 and September 2022. Consenting subjects participated in the research by accessing the QR code provided in the recruitment pamphlet via the internet.

Data collection was undertaken using a dedicated smartphone application for the data items and timings, as described in Table 2. The timings of data collection including voice recordings were daily during the recuperation period and, as a follow-up, one month, three months, and 6 months after the end of the recuperation period.

Although the number of participants registered during the recruitment period was 659, excluding participants who did not meet the participation criteria, such as being underage or having invalid data registration, or who withdrew participation during the research, the final number of participants was 581. Because the strength of infectivity, severity rate, and symptom characteristics differ depending on the type of COVID-19 mutation [16,17], an analysis was performed by differentiating the wave of infection (6th wave and beyond, 2022 onwards) caused by the omicron variant, which was known to be highly infectious compared to the previous variants of COVID-19 and the wave of infection before that (5th wave, up until 2021). Table 3 shows the distribution of the age of participants by the period of infection.

Moreover, Table 4 shows the aggregation results of the responses obtained from the questionnaire surveys conducted during the recuperation period of COVID-19 infection. Note that, depending on the participant, the count of questionnaire surveys differed, and, accordingly, the results shown here are for the responses obtained from the first questionnaire survey. As observed here, the percentage of participants exhibiting the well-known omicron variant characteristic symptoms of cough, runny nose, and sore throat was higher for the participants corresponding to the 6th wave and beyond than those corresponding to the 5th wave and prior.

### 2.3. Subject Classification and Extraction for Machine Learning

We divided the participants into two categories based on the symptoms of COVID-19, “mild illness” and “moderate illness I”, where the participants exhibiting the symptoms of “have respiratory distress” or “SpO2 ≤ 95%” were classified as “moderate illness I”. For the training of the machine learning models, age and sex were matched between the 5th wave and the 6th wave (see Table 3). Further, there were no participants used for the analysis who were classified as “moderate illness II” based on the symptoms of “SpO2 ≤ 93%” at the time of recording.

### 2.4. Voice Recording

The voice recording was conducted for three types of sustained vowels using the participant’s smartphone, with a dedicated application installed for the recording function. Table 5 shows a description of the phrases used for the voice recording.

### 2.5. Voice Analysis

The voice recordings were collected under various conditions, such as different smartphone models and microphone devices used by the study participants for recording, the positional relationship between the mouth and the microphone, the volume of the voice when speaking, and the surrounding noise and reverberations. Accordingly, a subjective evaluation of the sound quality was performed, and voices with good recording conditions were selected.

Subsequently, using only the voice recordings with good recording conditions, the speech features were extracted for each phrase using openSMILE. Here, based on the large openSMILE emotion feature set, 13,998 types of speech features were extracted by adding the analysis speech features.

To prevent overfitting while performing ML, good discriminative features were selected in advance. First, we divided the symptoms of COVID-19 patients into two categories: asymptomatic or mild illness, and moderate illness 1, creating a dummy variable. Subsequently, using this dummy variable as the dependent variable, we excluded features with no correlation with the dependent variable (|R| < 0.2) and those with a high correlation coefficient between the speech features (|R| > 0.9). Moreover, null importance was used for feature selection with 1000 bootstraps.

Five-fold cross-validation was used for LightGBM training, and the prediction performance was evaluated on the test set based on the sensitivity, specificity, accuracy, and AUC. The Optuna algorithm was used to tune the model hyperparameters [18]. The LightGBM parameter settings after tuning are described in Table 6.

Note that Microsoft Excel Office365 was used in the statistical analysis and Python 3.10 [19], and other related libraries were used in ML and feature selection using null importance.

## 3. Results

### 3.1. Selection of Voice Data for Analysis

To prevent bias due to different evaluators in the subjective evaluation of sound quality, one specific evaluator performed a subjective evaluation of all the voice data. The seven categories of subjective evaluation were ① normal, ② noisy (low), ③ noisy (high), ④ cough sound, ⑤ issues with volume (low/high), ⑥ short, sustained vowel duration, and ⑦ other issues. For the analysis, only category ① normal data were selected to avoid the effects of noise and sudden changes in sound, such as coughing, affecting the analysis results.

After the subjective evaluation and age/sex matching, the amount of subject data used in ML is shown in Table 7.

### 3.2. Feature Selection

Using the 13,998 speech features extracted using openSMILE, with each of the three types of sustained vowel sounds, feature selection based on the correlation coefficient with the response (dependent) variable and feature selection based on the correlation coefficient between the explanatory (independent) variables were performed. As a result, 57 speech features for /Ah/, 77 speech features for /Eh/, and 133 speech features for /Uh/ were selected.

Speech feature selection using null importance resulted in 5 features for /Ah/, 8 features for /Eh/, and 16 features for /Uh/ (Table 8). Examination of the features for /Ah/ revealed that features related to MFCC (Mel-frequency Cepstral-Coefficients), which is also used in the field of speech recognition as a noise-resistant speech feature, features related to auditory spectrum, and those related to magnitude spectrum were selected.

### 3.3. Validation

**Figure 1:**
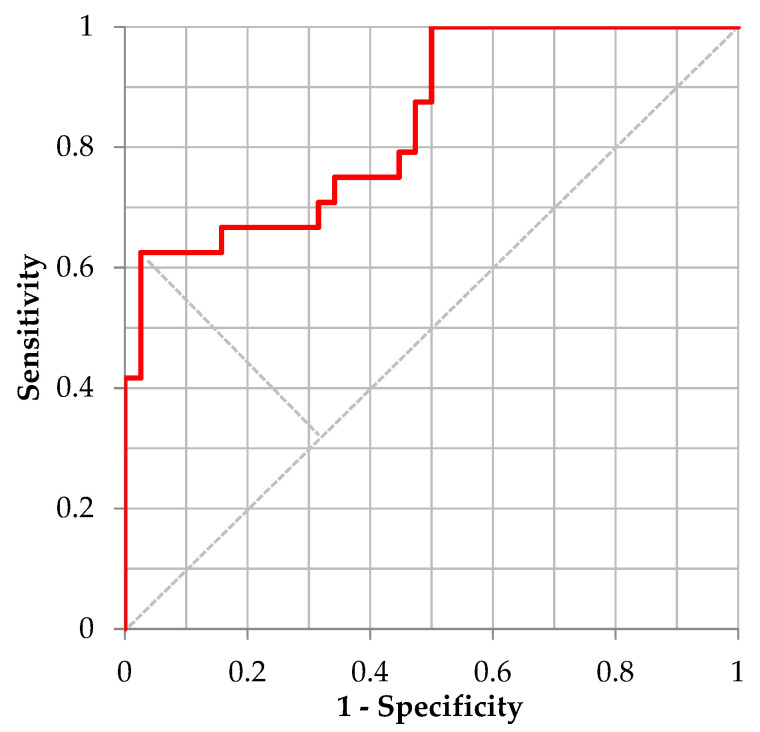
ROC curve distinguishing “Moderate Illness 1” using the learning model of /Ah/.

**Figure 2:**
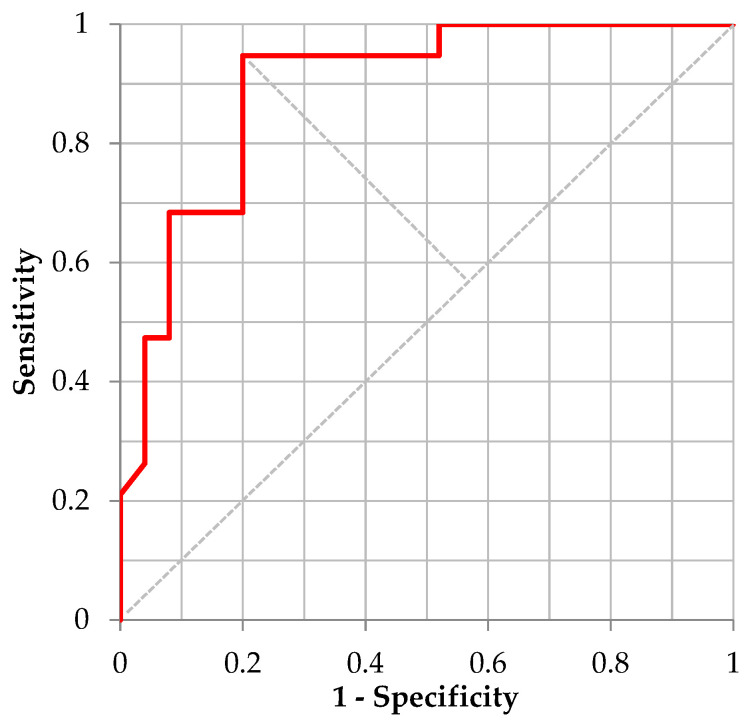
ROC curve distinguishing “Moderate Illness 1” using the learning model of /Eh/.

**Figure 3:**
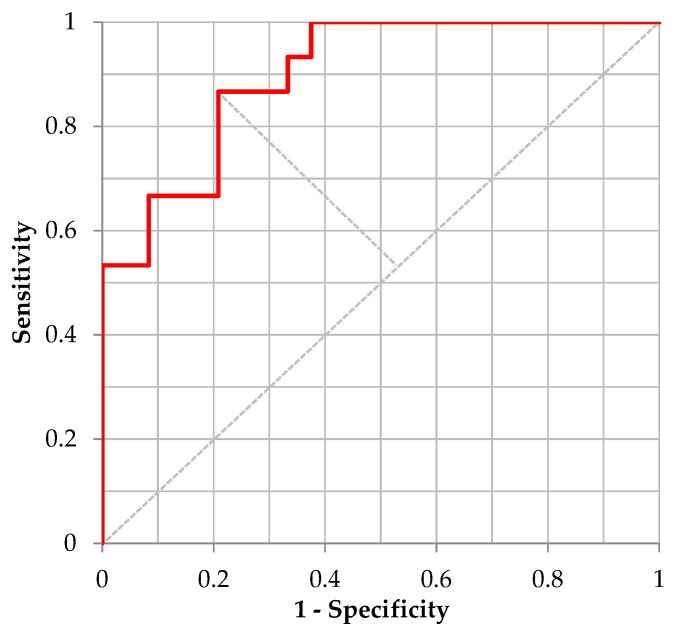
ROC curve distinguishing “Moderate Illness 1” using the learning model of /Uh/.

For the validation of distinguishing “Moderate Illness 1” using the ML model for /Ah/ utilizing 5-fold cross-validation, Table 9 shows the confusion matrix defined by the cutoff point value from the Youden’s Index and Figure 1 shows the corresponding ROC curve. The performance of the trained model for /Ah/, sensitivity, specificity, and accuracy was 0.625, 0.974, and 0.839, respectively. The corresponding AUC was 0.8399. For distinguishing “Moderate Illness 1” using the ML model for the sustained vowel /Eh/, Table 10 shows the confusion matrix defined by the cutoff point value from the Youden’s Index and Figure 2 shows the corresponding ROC curve. Regarding the performance of the trained model for /Eh/, the values of sensitivity, specificity, accuracy, and AUC were 0.947, 0.800, 0.864, and 0.8937, respectively. For distinguishing “Moderate Illness 1” using the ML model for sustained vowel /Uh/, Table 11 shows the confusion matrix defined by the cutoff point value from the Youden’s Index and Figure 3 shows the corresponding ROC curve. Regarding the performance of the trained model for /Uh/, the values of sensitivity, specificity, accuracy, and AUC were 0.867, 0.792, 0.821, and 0.9000, respectively.

**Figure 4:**
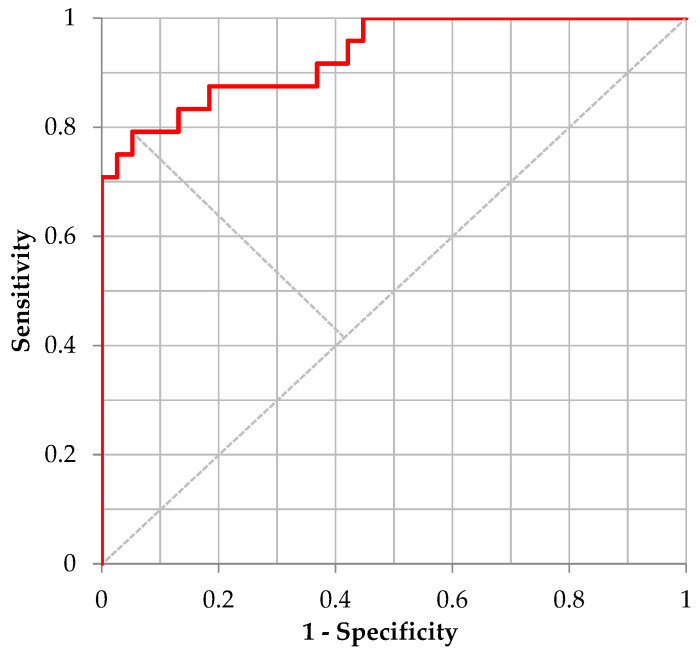
ROC curve for /Ah/+/Eh/+/Uh/.

Moreover, for distinguishing “Moderate Illness 1” using the ML model for sustained vowels /Ah/, /Eh/, and /Uh/ in combination, Table 12 shows the confusion matrix defined by the cutoff point value from the Youden’s Index and Figure 4 shows the corresponding ROC curve. Regarding the performance of the trained model for /Ah/, /Eh/, and /Uh/, the sensitivity, specificity, accuracy, and AUC were 0.792, 0.947, 0.887, and 0.9320, respectively.

## 4. Discussion

The results suggest that the generated model using the sustained vowels /Ah/, /Eh/, and /Uh/ is correctly functioning in distinguishing asymptomatic or mild illness symptoms and moderate illness I symptoms associated with COVID-19 infection. Using only one sustained vowel, the predictive performance exceeded an accuracy of 0.82 and AUC of 0.83. Using the combination of the three sustained vowels, the performance exceeded an accuracy of 0.88 and AUC of 0.93. These findings suggest that COVID-19-infection-associated impairments in the vocal organs, such as the throat, trachea, and airways, likely affect the voice, exhibited as vocal symptoms.

We believe that this study does not depend on the mother tongue of the patient, since we use only language-independent sustained vowels as analysis phrases. Moreover, since it does not analyze spontaneous speech, such as in interviews and dialogues, it can be easily conducted by one person without a dedicated measurer.

On the other hand, while we used only the voice data classified as “normal” based on the subjective evaluation of the voice data quality (Table 13), in the voice data recordings, we identified various issues, such as reverberation due to the recording environment, distance from the microphone, mixing of other people’s voices and noise, differences in microphone performance, recording problems due to processing failure of the smartphone, filter processing installed in the smartphone, and the content of the speech differing from the instructions. In addition to recording quality problems, anomalous numerical values that were possibly generated by operational errors during data registration were also confirmed. Moreover, there were cases where coughing, which is one of the symptoms of the COVID-19 infection, was included in the recorded voice. Such cases were excluded from the analysis, because the instantaneous changes in sound pressure and the included frequencies are different from normal vocalizations.

As mentioned above, studies to judge the existence of COVID-19 infection using cough sounds have been conducted [4,6,8]. However, we believe that it is difficult to collect accurately labeled cough sounds, since there are numerous factors that need to be separated, such as diseases other than COVID-19 infection, differences in the causes of cough, such as aspiration and dryness of saliva and foreign substances, and differences between an intentional cough and non-intentional cough.

One advantage of collecting speech and label information from research participants via the Internet is that it is possible to remotely collect data from a large number of participants. However, as described above, the quality and accuracy of the collected and recorded voice data depend on the participants. Accordingly, we believe that the data quality can be improved by implementing measures, such as evaluating the noise level and recording volume during recording and devising ways for displaying alerts and prompting re-recording when problems are identified, and making it impossible to register anomalous values when registering basic information and symptom responses.

Unlike the neuropsychiatric voice changes we have studied so far, we believe that the changes in voice associated with illness symptoms in this study capture additional acoustic changes in the voice, such as changes in the vocal tract due to inflammation of the pharynx and a decrease in the expiratory volume due to pneumonia. However, the underlying mechanism of such changes has not yet been adequately elucidated. Elucidating the mechanism of changes in voice is a topic of future research. In this study, we predicted the category of symptoms associated with COVID-19 infection using voice recordings of such patients. We believe that, in the future, judging whether a patient is infected with COVID-19 or not and building a model using robust speech features that are not easily affected by recording conditions are necessary.

A limitation of this study is that potential confounding factors, such as age, gender, and target period (5th or 6th wave), are not used in LightGBM model training. Specifically, it is commonly known that some acoustic features (e.g., fundamental frequency) are different between males and females or among ages [20]. Although we used age- and sex-matched data to minimize such confounder effects, and some of the acoustic features we used could be robust to such differences (e.g., MFCC features [21]), we cannot rule out the possibility of bias caused by these factors. Another potential confounder is the condition of the voice before COVID-19 infection, so it is not possible to know whether the condition of the voice is related to an acute infection or if it is a chronic condition of a voice disorder from another etiology (after surgery, unilateral vocal fold paralysis, etc.).

## 5. Conclusions

In this paper, we studied the efficacy of using speech features for judging the changes in the symptoms associated with COVID-19 infection. We collected voice recordings from the participants who were infected with COVID-19 and selected the voice data for analysis considering various factors, such as the age, group at the time of infection, sex, symptoms, and recording conditions. Subsequently, we extracted 13,998 speech features using openSMILE with the selected data. For the learning strategy, we selected features using statistical analysis and utilized the feature selection method of null importance, and, for the ML algorithm, we used LightGBM and validated the model performance using 5-fold cross-validation. Using the model for predicting “asymptomatic or mild illness” and “moderate illness I” using three types of sustained vowel sounds of /Ah/, /Eh/, and /Uh/, we were able to achieve high performance, demonstrated by an accuracy of 0.88 and AUC of 0.93.

We believe that the validation of the effects of noise and recording conditions and building a model that is robust enough against the effects of recording conditions are some future topics for further studies.

## Figures and Tables

**Table 1:** Classification of the severity of symptoms associated with COVID-19 infection.

Severity	Oxygen Saturation	Clinical State
Mild illness	SpO_2_ ≥ 96%	No respiratory symptom (no findings consistent with pneumonia) or Cough only without any shortness of breath (no findings consistent with pneumonia)
Moderate illness I No respiratory failure	93% < SpO_2_ < 96%	Shortness of breath and pneumonia findings
Moderate illness II Respiratory failure	SpO_2_ ≤ 93%	Oxygen administration required
Acute		ICU admission or Ventilator needed

**Table 2:** Data collected from the participants.

Timing	Item	Description
Join	Participation	Explaining the research outline and obtaining consent
	Basic Data	Sex, Age, Symptom onset date, Diagnosis confirmation date, Treatment start date
At the time of participation in research	Vitals data	Body temperature, Blood oxygen saturation
	Questionnaire	Change of symptoms, Symptomatic or not, Respiratory distress, Taste/olfactory disorder, Cough/sputum, Chest pain, Runny nose/nasal congestion, Sore throat, Nausea/vomiting, Diarrhea, Appetite, Fatigue, Headache, Joint pain, Rash, Red eyes
	Voice recording	Three sustained vowels
During treatment (recuperation)	Questionnaire	Confirmation on reason for treatment end (discontinuation)
Follow up (1 month, 3 months, and 6 months after the end of the recuperation period)	Vitals data	Body temperature, Blood oxygen saturation (for possible subjects only)
	Questionnaire	Change of symptoms, Symptomatic or not, Respiratory distress, Taste/olfactory disorder, Cough/sputum, Chest pain, Runny nose/nasal congestion, Sore throat, Nausea/vomiting, Diarrhea, Appetite, Fatigue, Headache, Joint pain, Rash, Red eyes
	Voice recording	Three sustained vowels

**Table 3:** Distribution of age of the participants. The number in parentheses represents the counts of the participants in the sex- and age-matched groups, which is extracted to build machine learning models.

Target Period	Age	Male	Male Age (Mean ± SD)	Female	Female Age (Mean ± SD)
5th wave and prior (~31 December 2021)	20–29	13 (1)	37.5 ± 12.1	19 (9)	32.0 ± 11.0
	30–39	13 (6)		3 (0)	
	40–49	9 (5)		3 (2)	
	50–59	7 (4)		5 (3)	
	60–69	0 (0)		0 (0)	
	70–79	1 (1)		0 (0)	
	80–89	0 (0)		0 (0)	
	subtotal	43 (17)	-	30 (14)	-
6th wave and beyond (1 January 2022~)	20–29	30 (1)	46.3 ± 12.9	54 (9)	
	30–39	44 (6)		67 (0)	
	40–49	70 (5)		70 (2)	
	50–59	55 (4)		59 (3)	41.9 ± 12.6
	60–69	35 (0)		14 (0)	
	70–79	7 (1)		3 (0)	
	80–89	0 (0)		1 (0)	
	subtotal	241 (17)	-	267 (14)	-
All periods	Total	284 (34)	45.0 ± 13.2	297 (28)	40.9 ± 12.8

**Table 4:** Percentage of participants exhibiting each of the specific symptoms (aggregation of the responses obtained in the first questionnaire).

Symptom	5th Wave and Prior (~31 December 2021)	6th Wave and Beyond (1 January 2022~)
SpO_2_ ≤ 95%	9 (12.3%)	34 (6.7%)
Body temperature ≥ 37.5 °C	7 (9.6%)	22 (4.3%)
Symptomatic	44 (60.3%)	386 (76.0%)
Respiratory distress	13 (17.8%)	96 (18.9%)
Taste/olfactory disorder	14 (19.2%)	77 (15.2%)
Cough/sputum	37 (50.7%)	366 (72.0%)
Chest pain	9 (12.3%)	78 (15.4%)
Runny nose/nasal congestion	27 (37.0%)	299 (58.9%)
Sore throat	21 (28.8%)	270 (53.1%)
Nausea	9 (12.3%)	25 (4.9%)
Diarrhea	16 (21.9%)	75 (14.8%)
Loss of appetite	21 (28.8%)	111 (21.9%)
Fatigue	28 (38.4%)	215 (42.3%)
Headache	20 (27.4%)	147 (28.9%)
Joint pain	15 (20.5%)	95 (18.7%)
Rash	3 (4.1%)	17 (3.3%)
Red eyes	5 (6.8%)	24 (4.7%)
Total	73	508

**Table 5:** Phrases used for the voice recording.

No.	Contents
Phrase01	/Ah/
Phrase02	/Eh/
Phrase03	/Uh/

**Table 6:** Parameter settings for the LightGBM classifiers.

Parameters	/Ah/	/Eh/	/Uh/
objective	binary	binary	binary
metric	binary_logloss	binary_logloss	binary_logloss
learning rate	0.01	0.01	0.01
lambda_l1	2.53 × 10^−6^	0.00	0.00
lambda_l2	6.50 × 10^−3^	0.00	0.00
num_leaves	31	31	31
feature fraction	0.42	0.40	0.80
bagging_fraction	0.84	1.00	1.00
bagging_freq	5	0	0
min_child_samples	10	10	5

**Table 7:** The amount of subject data used for analysis.

	Severity	
Target Period	Mild Illness	Moderate Illness I
5th wave and prior	19	12
6th wave and beyond	19	12
All periods	38	24

**Table 8:** Selected features.

Phrase	Selected Features with Rank_Importance
/Ah/	1. mfcc_sma(10)_upleveltime50
	2. audSpec_Rfilt_sma_de(16)_mtmAmpStddevRel
	3. pcm_fftMag_spectralMinPos_sma_de_peakRangeRel
	4. mfcc_sma(11)_max
	5. audSpec_Rfilt_sma_de(11)_rightctime
/Eh/	1. pcm_fftMag_fband90-180_sma_de_rightctime
	2. mfcc_sma(3)_downleveltime75
	3. mfcc_sma(8)_downleveltime90
	4. pcm_fftMag_spectralRollOff90.0_sma_de_peakRangeAbs
	5. audSpec_Rfilt_sma_de(1)_stddevFallingSlope
	6. audSpec_Rfilt_sma_de(1)_lpc3
	7. audSpec_Rfilt_sma_de(1)_lpc4
	8. mfcc_sma(14)_percentile98.0
/Uh/	1. audSpec_Rfilt_sma(13)_lpc4
	2. pcm_fftMag_spectralHarmonicity_sma_de_minRangeRel
	3. audSpec_Rfilt_sma(12)_lpc4
	4. audSpec_Rfilt_sma_de(19)_numPeaks
	5. audSpec_Rfilt_sma(21)_ptpAmpStddevAbs
	6. pcm_fftMag_spectralMinPos_sma_de_ptpAmpMeanAbs
	7. audSpec_Rfilt_sma(16)_lpc4
	8. audSpec_Rfilt_sma_de(11)_ptpAmpMeanRel
	9. audSpec_Rfilt_sma(19)_upleveltime25
	10. audSpec_Rfilt_sma_de(8)_numSegments
	11. pcm_fftMag_spectralCentroid_sma_de_leftctime
	12. mfcc_sma_de(11)_quartile1
	13. voicingFinalUnclipped_sma_de_lpc4
	14. pcm_fftMag_psySharpness_sma_de_leftctime
	15. mfcc_sma(9)_linregc2
	16. mfcc_sma_de(14)_ptpAmpStddevAbs

**Table 9:** Confusion matrix of /Ah/.

		Predicted Group	
		Mild Illness	Moderate Illness I
Actual group	Mild Illness	37	1
	Moderate Illness I	9	15

**Table 10:** Confusion matrix of /Eh/.

		Predicted Group	
		Mild Illness	Moderate Illness I
Actual group	Mild Illness	20	5
	Moderate Illness I	1	18

**Table 11:** Confusion matrix of /Uh/.

		Predicted Group	
		Mild Illness	Moderate Illness I
Actual group	Mild Illness	19	5
	Moderate Illness I	2	13

**Table 12:** Confusion matrix of /Ah/, /Eh/, and /Uh/.

		Predicted Group	
		Mild Illness	Moderate Illness I
Actual group	Mild Illness	36	2
	Moderate Illness I	5	19

**Table 13:** Judgement results of subjective evaluation of all voice data and questionnaire (responses).

Target Data	Judgement	Data Count		
		/Ah/	/Eh/	/Uh/
Voice	Normal	983	1122	1073
	Noisy (low)	447	474	544
	Noisy (high)	379	367	507
	Cough	12	13	9
	Volume	526	352	207
	Short, sustained duration	25	16	16
	Other issues	224	248	235
	No recording	464	468	469
Questionnaire	Normal value	2975		
	Anomalous value	85		
	Total registrations	3060		

## Data Availability

The data are not publicly available due to personal information contained within.
